# Increased Oxidative Stress in the Prefrontal Cortex as a Shared Feature of Depressive- and PTSD-Like Syndromes: Effects of a Standardized Herbal Antioxidant

**DOI:** 10.3389/fnut.2021.661455

**Published:** 2021-04-15

**Authors:** Johannes de Munter, Dmitrii Pavlov, Anna Gorlova, Michael Sicker, Andrey Proshin, Allan V. Kalueff, Andrey Svistunov, Daniel Kiselev, Andrey Nedorubov, Sergey Morozov, Aleksei Umriukhin, Klaus-Peter Lesch, Tatyana Strekalova, Careen A. Schroeter

**Affiliations:** ^1^Department of Psychiatry and Neuropsychology, School for Mental Health and Neuroscience, Maastricht University, Maastricht, Netherlands; ^2^Laboratory of Psychiatric Neurobiology, Institute of Molecular Medicine and Department of Normal Physiology, Sechenov First Moscow State Medical University, Moscow, Russia; ^3^Rehabilitation Research Unit of Clinic of Bad Kreuzbach, Bad Kreuzbach, Germany; ^4^PK Anokhin Research Institute of Normal Physiology, Moscow, Russia; ^5^Ural Federal University, Yekaterinburg, Russia; ^6^Institute of Translational Biomedicine, St. Petersburg State University, St. Petersburg, Russia; ^7^Neuroscience Program, Sirius University, Sochi, Russia; ^8^School of Biological and Medical Physics, Moscow Institute of Physics and Technology, Moscow, Russia; ^9^Institute for Translational Medicine and Biotechnology, Preclinical Research Center of Sechenov First Moscow State Medical University, Moscow, Russia; ^10^Federal Budgetary Institute of General Pathology and Pathophysiology, Moscow, Russia; ^11^Division of Molecular Psychiatry, Center of Mental Health, University of Würzburg, Würzburg, Germany; ^12^Department of Preventive Medicine, Maastricht Medical Center Annadal, Maastricht, Netherlands

**Keywords:** antioxidant nutrients, oxidative stress, depression, post-traumatic stress disorder, pro-inflammatory cytokines, prefrontal cortex, forced swimming, mice

## Abstract

Major depression (MD) and posttraumatic stress disorder (PTSD) share common brain mechanisms and treatment strategies. Nowadays, the dramatically developing COVID-19 situation unavoidably results in stress, psychological trauma, and high incidence of MD and PTSD. Hence, the importance of the development of new treatments for these disorders cannot be overstated. Herbal medicine appears to be an effective and safe treatment with fewer side effects than classic pharmaca and that is affordable in low-income countries. Currently, oxidative stress and neuroinflammation attract increasing attention as important mechanisms of MD and PTSD. We investigated the effects of a standardized herbal cocktail (SHC), an extract of clove, bell pepper, basil, pomegranate, nettle, and other plants, that was designed as an antioxidant treatment in mouse models of MD and PTSD. In the MD model of “emotional” ultrasound stress (US), mice were subjected to ultrasound frequencies of 16–20 kHz, mimicking rodent sounds of anxiety/despair and “neutral” frequencies of 25–45 kHz, for three weeks and concomitantly treated with SHC. US-exposed mice showed elevated concentrations of oxidative stress markers malondialdehyde and protein carbonyl, increased gene and protein expression of pro-inflammatory cytokines interleukin (IL)-1β and IL-6 and other molecular changes in the prefrontal cortex as well as weight loss, helplessness, anxiety-like behavior, and neophobia that were ameliorated by the SHC treatment. In the PTSD model of the modified forced swim test (modFST), in which a 2-day swim is followed by an additional swim on day 5, mice were pretreated with SHC for 16 days. Increases in the floating behavior and oxidative stress markers malondialdehyde and protein carbonyl in the prefrontal cortex of modFST-mice were prevented by the administration of SHC. Chromatography mass spectrometry revealed bioactive constituents of SHC, including D-ribofuranose, beta-D-lactose, malic, glyceric, and citric acids that can modulate oxidative stress, immunity, and gut and microbiome functions and, thus, are likely to be active antistress elements underlying the beneficial effects of SHC. Significant correlations of malondialdehyde concentration in the prefrontal cortex with altered measures of behavioral despair and anxiety-like behavior suggest that the accumulation of oxidative stress markers are a common biological feature of MD and PTSD that can be equally effectively targeted therapeutically with antioxidant therapy, such as the SHC investigated here.

## Introduction

A large portion of the human population is chronically exposed to stress. Nowadays, given the dramatically developing COVID-19 situation globally, a degree of stress can become devastating and even traumatic in most countries (1). This is prone to cause a high incidence of neuropsychiatric disorders, including major depression (MD), posttraumatic stress disorder (PTSD), and other stress-related conditions (2). Apart from the mentioned medical problems resulting from the COVID-19 pandemic, its devastating economic consequences can hamper the use of costly antidepressants and sedatives, particularly in countries with limited medical care. These factors necessitate further development of inexpensive and effective alternatives to current therapies and prevention approaches (3). Herbal medicine appears to be a reasonable treatment of neuropsychiatric disorders that is more affordable and with fewer side effects than classic pharmaca. With this problem, there is a need for safe herbal remedies for these socially significant diseases. As such, the importance of addressing this issue in studies using advanced animal models of MD and PTSD for society cannot be overstated.

Oxidative stress and inflammation attract increasing attention as important mechanisms of neuropsychiatric disorders, which are often targeted with herbal medicine (4, 5). A variety of herbal extracts and herbal compositions with stress-reducing properties were previously shown to exert anti-inflammatory and antioxidant effects, being effective in patients with stress-related and other disorders. For example, ginger phenolics decrease lipid peroxidation and oxidative stress neuronal damage in rats (6), vanilla suppresses free radical production in a mouse model of cancer (5), and polysaccharides from red seaweed suppress expression of tumor necrosis factor (TNF) receptor–associated factor-6 in a model of LPS-induced toxicity (7). Our recent studies with an herbal composition containing five herbs shows beneficial action on anxiety-like behavior, hippocampal malondialdehyde (MDA) concentration and expression of AMPA receptors in mice subjected to the “emotional” ultrasound stress (US) model (8).

Currently, the role of oxidative stress and associated neuroinflammation in MD and PTSD is well established (9–13). Increased production of free radicals and/or reduced antioxidant defenses under challenged conditions result in excessive levels of free radicals in the brain, leading to mitochondrial dysregulation, microglia activation, and neuronal death (14–17). These mechanisms are suggested to play a key role in helplessness, anxiety, and inappropriate retention of aversive memories (9, 10, 18–21).

An increasing body of data suggests modulatory interconnections between oxidative stress and increased inflammation as well as overexpression of glycogen synthase kinase 3 (GSK-3) cascade during depressive and PTSD-related conditions (22–24). Augmented production of inflammatory cytokines is found to trigger microglial activation that further upregulates their expression (25–27), leading to suppressed neurogenesis, survival, migration, and recruitment of new neurons and eventually neuronal dysfunction (28–31). Moreover, microglial activation further promotes oxidative stress in the brain (14–17, 32). Pro-inflammatory cytokines can inhibit production of anti-inflammatory cytokines, such as interleukin-15 (IL-15) and phosphorylated Akt kinase, functional antagonists of major pro-apoptotic/cellular stress molecules, GSK-3β and GSK-3α (12, 33). Increased expression and protein activities of GSK-3β are well-documented correlates of neuronal degeneration (33–35).

A number of oxidative stress markers are found to be upregulated in patients with various forms of affective abnormalities and PTSD (9, 36–39). For instance, increased plasma levels of oxidative stress marker 8-hydroxy-2'-deoxyguanosine is shown in patients with agitated depression (39). Positive correlations between blood levels of C-reactive protein, pro-inflammatory cytokines (such as IL-1β, IL-6, and TNF), and cyclooxygenase-1 (COX-1) and the enhanced learning of aversive memories are demonstrated in patients with PTSD (11, 40, 41). Brain overexpression of pro-inflammatory cytokines and GSK-3 is associated with treatment resistance in depressed patients with comorbidity for PTSD (42–44). Upregulation of GSK-3 is shown to be implicated in the processing of aversive memories during PTSD (45) and emotional and cognitive dysregulation during depressive syndrome (46).

Although oxidative stress is frequently the target mechanism of herbal medicine in numerous studies, it has to be noted that the majority of reported translational approaches rely on other than established models of psychiatric disorders mimicking MD- and PTSD-like features. Recently, we proposed new mouse paradigms of MD and PTSD in which the role of oxidative stress and neuroinflammation as well as normalizing effects of antioxidants were demonstrated. A new mouse paradigm of MD, the model of US, was designed to mimic emotional stress in rodents instead of generally used chronic stress procedures that are based on physical stressors (8, 47, 48). We also established a novel mouse paradigm of enhanced contextual conditioning of adverse memories, the modified forced swim model (modFST), in which the classic two-day swimming test is followed by an additional swim test on day 5 (24, 49, 50). Increased floating behavior and overexpression of GSK-3β in the prefrontal cortex that are exhibited during the delayed swim session were found to be context-dependent and reversible by a pretreatment with antidepressant and antioxidant compounds (24, 51). These features validate the modFST as a PTSD model in contrast to a routine swim test. In both paradigms, brain increases of oxidative stress markers and pro-inflammatory cytokines are shown (48–50, 52).

In the present study, we sought to study oxidative stress changes in the prefrontal cortex of mice exposed to US and modFST also under conditions of chronic administration of an antioxidant standardized herbal cocktail (SHC) to investigate adjusting behavioral, neuroinflammatory, and stress-related molecular changes in these paradigms. We chose to study molecular changes in the prefrontal cortex as this brain structure is implicated in the pathogenesis of both MD and PTSD (45, 53) and because contextual potentiation of floating behavior in the modFST correlates with stress markers, such as GSK-3β activity, in this but not other brain areas (24).

We studied the accumulation in the prefrontal cortex of MDA, a classic oxidative stress marker (54) that was not yet investigated yet in the US and modFST models. It is detectable in the smallest amount of tissue among similar markers, allowing for individual analysis of each sample and a clearer picture of its correlation with other readouts, such as behavioral measures, which is one the main purposes of the current work. In addition, concentrations of protein carbonyl, another established marker of oxidative stress (55, 56), were studied.

With the US model, BALB/c mice were subjected to ultrasound frequencies of 16–20 kHz, mimicking rodent sounds of anxiety and despair, which are randomly alternating with “neutral” frequencies of 25–45 kHz, for three weeks, as described elsewhere (47). Cohorts of mice concomitantly received antioxidant treatment with SHC or vehicle; mice were studied for helplessness in the forced swim test, anxiety-like behavior, and concentrations of protein carbonyl and MDA in the prefrontal cortex. Congruently, in the modFST model, mice were pretreated with SHC or vehicle for 16 days and investigated for floating, anxiety-like, and open field behaviors and concentrations of protein carbonyl and MDA.

Additionally, in view of the changes observed in these studies, C57BL/6 mice that were not used in the US model so far were exposed to the US protocol and antioxidant therapy as in the study on BALB/c mice and investigated for the described behaviors, gene and protein expression of oxidative stress-related pro-inflammatory cytokines IL-1β and IL-6, anti-inflammatory cytokine IL-15, and markers of cellular stress GSK-3β and GSK-3α as well as counteracting this cascade Akt/Akt phosphorylated kinase in the prefrontal cortex. Among numerous anti-inflammatory cytokines, IL-15 was studied because of its unique role in periphery-brain mechanisms of immune microglial activation (57), oxidative stress mechanisms, and a demonstration of Akt- and GSK-3β-mediated regulation of cellular immune response and cell survival (58, 59). Correlation analysis was performed between molecular and behavioral measures.

## Methods

### Animals

Experiments were performed on male BALB/c and C57BL/6 mice that were 3 months old; animals were provided by a provider licensed by Charles River (http://www.spf-animals.ru/about/providers/animals). Mice were housed individually in standard plastic cages (27x22x15 cm) and maintained on a reversed 12-h light/dark cycle under controllable laboratory conditions (22 ± 1°C, 55% humidity, room temperature 22°C; lights were on at 19:00). Food and water were available ad libitum. All efforts were undertaken to minimize the potential discomfort of experimental animals. Experimental protocols conformed to directive 2010/63/EU, were compliant with ARRIVE guidelines (http://www.nc3rs.org.uk/arrive-guidelines), and were approved by local veterinarian committees (iCell2 METC Zuyderland Zuid, the Netherlands and MSMU #11-18-2018/2019).

### Study Design

**Figure 1:**
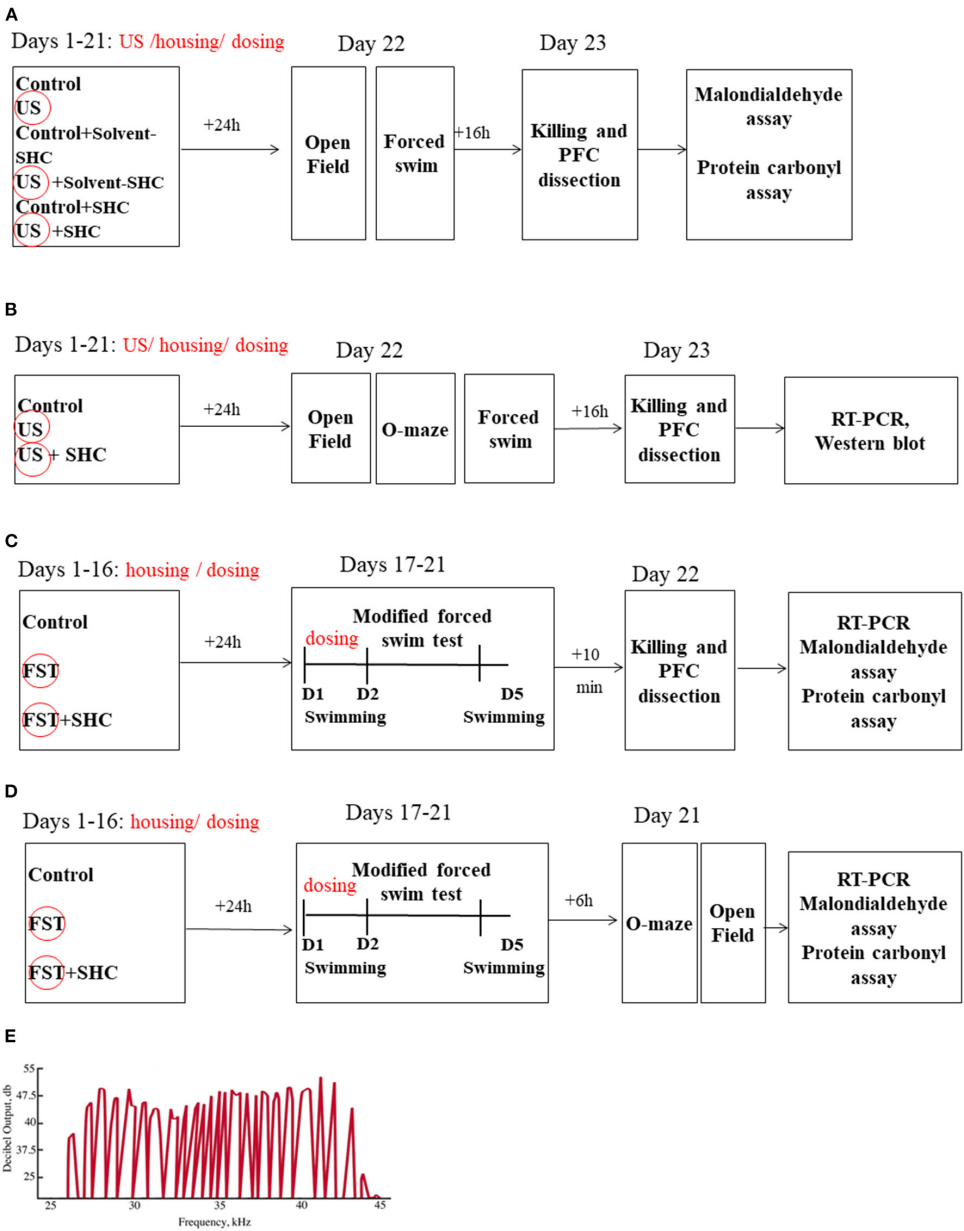
Study flow. **(A)** BALB/c mice remained unstressed or were submitted to US for three weeks; they were either nontreated or received SHC solvent or SHC (*n* = 7 per group). Thereafter, mice were weighed and studied for the open field and forced swim tests and consequently killed; their prefrontal cortex was dissected for the MDA and protein carbonyl assays. **(B)** Following the above-described US and SHC treatment, C57BL/6 mice (*n* = 8 per group) were weighed and scored for floating and open field and O-maze behaviors and consequently killed; their prefrontal cortex was dissected for the RT-PCR and Western blot examination of the expression of GSK-3β and GSK-3α, Akt/Akt-pSer473, IL-1β, IL-6, and IL-15. **(C)** C57BL/6 mice were nontreated or pretreated with SHC for 16 days and submitted to 2-day 6-min swimming sessions (days 1 and 2), followed by an additional session on day 5. They were killed 10 min after the last session together with nonmanipulated controls (*n* = 10 per group); their prefrontal cortex was dissected for MDA and protein carbonyl assays. **(D)** In the modFST, C57BL/6 mice were pretreated with SHC for 16 days and subjected to swimming sessions as described above (*n* = 7 in each group). They were studied for anxiety-like behavior in the O-maze and the open field and sacrificed 10 min after the last test for MDA assay of the prefrontal cortex. **(E)** The shape of the ultrasound signals was fluctuating, mimicking natural ultrasonic vocalizations of rodents. PFC, prefrontal cortex; FST, forced swim test; D, Day. US/FST-exposed groups are highlighted with a circle.

The experimental design and sequence of procedures are presented in Figures 1A–D. In the US model, BALB/c mice (*n* = 7 in each group) were unstressed or submitted to the US for three weeks; they were either nontreated or received SHC solvent or SHC, which was administered daily *per os* via a pipette as described (see below; Figure 1A). The ultrasound of alternating frequencies that mimics naturally emitted sounds of anxiety and despair and neutral sounds were applied as described; 24 h thereafter, mice were weighed and studied in the swim test and the open field; and 16 h later, mice were anesthetized and killed (see below) and their prefrontal cortex was dissected for the MDA and protein carbonyl assays (see below). As virtually no effects of SHC solvent were found in this experiment, vehicle was not used in other studies. In another US study, male three-month-old C57BL/6 mice (*n* = 8 in each group) were exposed to the US and SHC as in the previous experiment (Figure 1B). In 24 h, they were investigated in the swim test, the elevated O-maze, and the open field test. In 16 h, animals were killed and their prefrontal cortex was dissected for the RT-PCR and Western blot examination of the expression of GSK-3β and GSK-3α, Akt/Akt-pSer473, IL-1β, IL-6, and IL-15 (see below). In the modFST, C57BL/6 mice were pretreated with SHC for 16 days and subjected to two-day 6-min swimming sessions with a 24 h interval (days 1 and 2), followed by an additional session on day 5 (Figure 1C). They were killed 10 min after the last session simultaneously with nonmanipulated controls (*n* = 10 in each group) and their prefrontal cortex was dissected for MDA and protein carbonyl assays. The same treatment scheme was applied on a separate cohort of mice, which was studied for anxiety-like behavior in the O-maze and the open field, using the above-listed parameters, on day 5 of the experiment (*n* = 8 in each group; Figure 1D). Time for euthanasia was defined in previous studies with employed models that are also in accordance with other literature (24, 30, 48). The number of animals per group was selected based on current welfare regulations and former experience with the US and modFST paradigms and findings of interstrain differences in susceptibility to stress (24, 48, 60).

### Antioxidant Treatment With SHC

The drops were made out of herbs, spices, and dried fruits to match an SHC, an extract of clove, bell pepper, basil, pomegranate, nettle, and other plants that was designed as an antioxidant treatment (Table 1) diluted in 30% alcohol solvent, SHC solvent (kindly provided by Mr. R. Vendeville, Voorhout, The Netherlands).

**Table 1:** Composition of SHC and SHC solvent.

**Solution**	**SHC**	**SHC-solvent**
Composition	Pomegranate	Alcohol 30%, water
	Nettle	
	Clove Basil	
	Bell pepper	
	Turmeric	
	Alcohol 30%	

Solutions of the SHC or the SHC solvent were administered orally using a pipette for 21 days concomitantly with ultrasound emission in the US studies and during 16 days prior the start of the modFST experiment. In the latter study, the dosing was continued throughout days 1 and 2 of the test lasting, in total, 18 days. Solutions were dosed in a volume of 120 μl per day for each mouse during the morning hours. Gas chromatography mass spectrometry analysis of SHC was carried out (see Supplementary Material). Chromatography mass spectrometry revealed bioactive constituents of SHC, including D-ribofuranose; beta-D-lactose; and malic, glyceric, and citric acids (Table 2).

**Table 2:** Chemical composition of SHC of molecules with a content of >1%.

**Chemical component**	**Percentage, by dry weight**	**Chemical class**
Methyl α-D-Glucopyranoside	39.4%	Methyl monosaccharide
Methyl β-D-Galactopyranoside	9.5%	Methyl monosaccharide
D-Fructofuranose	5.5%	Monosaccharide
D-Ribofuranose	2.5%	Monosaccharide
β-D-Lactose	2.2%	Monosaccharide
D-Glucose	2.0%	Monosaccharide
Malic acid	3.2%	Organic acid
Glyceric acid	1.5%	Organic acid
Citric acid	1.0%	Organic acid

### Emotional US

We used ultrasound radiation of intensity of 50 ± 5 dB and variable frequencies in a 20–45 Hz range as described elsewhere (8, 47, 48). The intensity of the sound fluctuated at the range ±10% of the averaged value, i.e., ±5 dB. It was constantly emitted in a laboratory room to the experimental groups of animals using a random schedule of alternating frequencies via a manufactured device (Weitech, Wavre, Belgium). During the 10-min periods, ultrasound frequencies fluctuated at variable short time spans of ≤1 s (average frequency 70 ± 10 Hz). Thirty percent of the emission time consisted of frequencies 40–45 kHz, and 35% of the emission consisted of frequencies 20–25 and 25–40 kHz; the remaining 35% consisted of low frequencies (16–20 kHz). The selectivity of the adverse effects of low-frequency ultrasound in comparison with the potential general negative effects of a constant noise as well as pharmacological sensitivity of the US-induced behavioral changes and blood cortisol increases to fluoxetine and buspirone in mice were shown previously (47, 52). The shape of the ultrasound signals was fluctuating, mimicking natural ultrasonic vocalizations of rodents (Figure 1E).

### modFST

In the modFST, mice were first tested in a two-day swimming protocol on days 1 and 2 using a previously established method (61) and then additionally studied under the same conditions on day 5 (24). Treatment with SHC was continued on days 1 and 2. Mice were placed for 6 min in a transparent cylinder (Ø 17 cm) filled with water (+23°C, water height 13 cm, height of cylinder 20 cm, under subtle lighting). The parameters of floating behavior were scored as in the forced swim test; in addition, the number of floating episodes was measured. Data from day 5 were normalized to data from day 2 and expressed in percentage. This parameter was used as a previously established measure of potentiation of floating behavior resulting from repeated exposure to a context of forced swimming on day 5 (24, 50, 51).

### Behavioral Testing

All behavioral tests were carried out during the active period of the animals' light cycle (09:00–21:00); behavior was recorded and scored off-line. The experimenter was unaware of the treatment each animal had received. Behavioral equipment was thoroughly cleaned with water between each test.

#### Open Field Test

In the open field paradigm, animals were placed in a square gray plastic box (45 × 45 × 45 cm) illuminated with white light (25 lx) near the wall for 10 min (Open Science, Moscow, Russia). The number of rearings, a measure of novelty exploration, and the distance covered were scored as described elsewhere (48). The recordings were analyzed using the automated video-tracking Viewpoint software (Viewpoint, Lyon, France).

#### Forced Swim Test

The test was performed as described elsewhere (61). Mice were placed into a plastic transparent cylinder (Ø 17 cm) filled with water (+23°C, water height 13 cm, cylinder height 20 cm) under subtle lightning (light intensity 25 lx) for 6 min. Their parameters of floating behavior, defined by the absence of any directed movements of the animals' head and body, were scored off-line using the automated video-tracking Viewpoint software (Viewpoint, Lyon, France). The total time spent floating was evaluated for the entire duration of the test; the latency to float was recorded as well.

#### Elevated O-Maze

The apparatus (Open Science, Moscow, Russia) consisted of a circular path (runway width 5.5 cm, diameter 46 cm) that was placed 45 cm above the floor. Two opposing arms were protected by walls (closed area, height 10 cm), and the illumination strength was 25 Lux. The apparatus was placed on a dark surface to maintain control over lighting conditions during testing. Mice were placed in one of the closed-arm areas of the apparatus. Behavior was assessed using previously validated parameters during a 5-min observation period (62, 63). The latency of the first exit to the open compartments of the maze and the number of exits to the open arms were scored.

### Culling and Dissection of Prefrontal Cortex

Mice were terminally anesthetized with isoflurane inhalation for a subsequent material collection and then transcardially perfused with 10 mL ice-cold saline. Brain was dissected and prefrontal cortex was isolated and stored at −80°C until use for gene expression analysis. All procedures were carried out as described previously (24).

### Biochemical Assays

#### MDA Assay

We chose to use MDA concentration in the brain because it is an established parameter of lipid peroxidation (54) that is produced in high concentrations in the brain tissue (64), thus allowing obtaining a sufficient amount of tissue for other assays from the same animal. Concentrations of MDA were measured following Abcam ab118970 kit instructions (Abcam, Eugene, OR, USA). Briefly, the tissue was washed in cold PBS and homogenized in lysis solution, centrifuged at 13,000 g for 10 min. TBA reagent was added to a supernatant and incubated at 95°C for 60 min; the supernatant was analyzed at 532 nm in a 96-well-microplate as described elsewhere (8).

#### Protein Carbonyl Assay

Determination of protein carbonyls used the OxiSelect™ Protein Carbonyl Fluorometric Assay kit (Cell Biolabs, Inc., San Diego, USA). Glass–glass homogenization of frozen prefrontal cortex followed by sonification was performed on ice in 1 ml of 1 × sample diluent from the OxiSelect™ kit, centrifuged at 10,000 g for 5 min at 4°C, and the supernatant removed. The total protein concentration was adjusted to 1–10 mg/ml with 1 × sample diluent, and protein carbonyls were determined according to the guidelines of the manufacturer using the GloMax Multi Detection System (Promega, Madison, WI, USA) equipped with a fluorescence module (485/540 nm filter set). Results were normalized to protein concentration as described previously (55, 65).

### Quantitative Reverse Transcription Polymerase Chain Reaction Analysis (RT-PCR)

qRT-PCR was performed as described elsewhere (50), using CFX96 the Deep Well Real-Time PCR Detection System (Bio-Rad, Hercules, CA, USA) in a 10-μl reaction volume containing 5 μl of SYBR Green master mix (Bio-Rad Laboratories, Philadelphia, PA, USA), 3 μl of RNase-free water, 1 μl of cDNA, and 1 μl of specific forward and reverse primers at a concentration 20 pmol/μl. The initial denaturation step for qRT-PCR was set at 95°C for 4 min followed by 40 cycles of denaturation at 95°C for 20 s; annealing was at 54°C for 90 s. All samples were run in triplicate. Sequences of all primers used (Evrogen, Moscow, Russia) are listed in Supplementary Table 1.

### Western Blot Assay

Western blot assay was carried out as described elsewhere (48). Frozen tissue was treated with lysis buffer containing 20 mM of Tris-HCl (pH 7.5), 450 mM of NaCl, 1% solution of Triton X-100, 1 mM of EDTA, 1 mM of NaF, 1 mM of Na_3_VO_4_, and protease inhibitor (Roche Diagnostics, Indianapolis, IN, USA); 50 μl of buffer per 1 g of tissue was used. Samples were centrifuged at 16,000 rpm for 20 min at 4°C and supernatant was collected and stored until use at −20°C. Twenty-five micrograms of protein from each sample was mixed with 35 μl of Laemmli buffer. A sample of identical volume, comprising 26 μl of Laemmli buffer, 5 μl of Page Ruler, and 4 μl of Magic Mark (Sigma, Munich, Germany), was used as a reference. For electrophoresis, samples were diluted in a solution containing MiliQ H_2_O, 1.5 M of Tris Buffer (pH 8.8), 30% solution of Acrylamide, 10% solution of SDS Temed, and 10% solution of ammonium persulfate (APS). For the next step, a solution containing MiliQ H_2_O, 0.5 M of Tris Buffer (pH 8.8), 30% solution of Acrylamide, 10% solution of SDS Temed, 10% solution of APS, and gel (Sigma, Munich, Germany) was used. The percentage of gel solution was adjusted to the sizes of proteins of interest as follows: 20% for proteins of size of 4–40 kDa, 12.5% for proteins of the size of 40–70 kDa, 10% for proteins of the size of 70–100 kDa, and 7.5% for proteins larger than 100 kDa. A buffer containing 25 mM of Tris Base buffer, 192 mM of Glycine (Sigma, Mannheim, Germany), 10% solution of SDS, and MiliQ H_2_O (pH 8.3) was used for gel electrophoresis, which was carried out under the constant voltages of 80 and 130 V. A polyvinylidene difluoride (PVDF) membrane (9 x 6 cm, EMD Millipore, Billerica, MA, USA) was consequently incubated in a 99% methanol solution for 1 min (Brocacef, Amsterdam, the Netherlands), MiliQ H_2_O for 5 min, and a transfer buffer for 15 min. The latter contained 25 mM of Tris Base, 192 mM of glycine, 20% solution of methanol, and MiliQ H_2_O (pH 8.3). For the next step, a blot “transfer sandwich” was composed of a buffer-soaked sponge, consisting of two buffer soaked Whatman filter papers, gel, activated membrane, and ice-cold transfer buffer; the constant current of 300 mA was used for 2 h 30 min.

Thereafter, the membrane was treated with a 5% dry milk solution; the TBST, containing 50 mM of Tris-HCl (pH = 8.2); 150 mM of NaCl; and 0.05% solution of Tween 20 (Sigma, Munich, Germany) for 1 h at room temperature and subsequently incubated with primary antibodies (see Supplementary Table 2) at 4°C overnight, which was followed by the incubation with respective horseradish peroxidase-conjugated secondary (HRP) antibodies (Sigma-Aldrich, St. Louis, MO, USA) for 2 h at room temperature on a roller. The membrane was washed in TBST three times, 5 min each time, and placed on the plastic cover. Thereafter, the Western Bright^TM^ ECL kit (Advansta Inc, Menlo Park, CA, USA) was used according to the manufacturer's instructions. A relative optical density of immunoreactive protein bands was examined using ImageJ software (NIH, Bethesda, MD, USA). Results were normalized to the relative intensity of the β-tubulin band that was selected as a reference protein as described elsewhere (8, 66). Blots were stripped by incubation with Restore Western Blot stripping buffer (Thermo Scientific, Rockford, IL, USA) at room temperature for 15 min.

To normalize the data, the value of each protein of interest was expressed in percentage from the concentration value of β-tubulin, the reference protein. The choice of a reference protein was based on the previous observations in which its expression was found to vary moderately across various experimental conditions and the linear representation of its signal intensity was demonstrated as described elsewhere (48).

#### Determination of Protein Concentration

Protein concentration was quantified using the BCA protein assay kit (Pierce, Rockford, IL, USA). The working reagent was prepared in accordance with manufacturer instructions: 25 μl of each standard or sample preparation were pipetted into a microplate well, 200 μl of the working reagent was added to each well and mixed thoroughly on a plate shaker for 30 seconds and the assay was run in duplicate. The covered plate was incubated at 37°C for 30 min and cooled to room temperature for 10 min. The absorbance was measured at 562 nm in a Biotek Microplate Reader (Biotek Instruments, Winooski, VT, USA). The Ascent Software Program (Winooski, VT, USA) coupled to the microplate reader was used to calculate protein values based on comparing optical density readings of the experimental samples with those obtained from the standard curve; the blank value was subtracted from all other optical density readings. A standard curve was generated by plotting the average blank-corrected 562 nm measurements for each BSA standard vs. its concentration in μg/ml. Information on primary antibodies used in the Western blot assay is presented in Supplementary Table 2.

### Statistics

Data were analyzed using GraphPad Prism version 8.01 (San Diego, CA, USA). Two- and one-way ANOVAs were employed to perform four- and three-group comparisons, respectively, followed by Tukey's *post-hoc* test. The unpaired two-tailed *t*-test was applied for two-group comparisons. For correlation analysis, Pearson correlation was used; *p* < 0.05 was set as a level of significance. Data were presented as mean ± SEM or mean.

## Results

### Ultrasound Stress Induces Helplessness and MDA Accumulation: Effects That Are Ameliorated by Herbal Antioxidant Treatment

**Figure 2:**
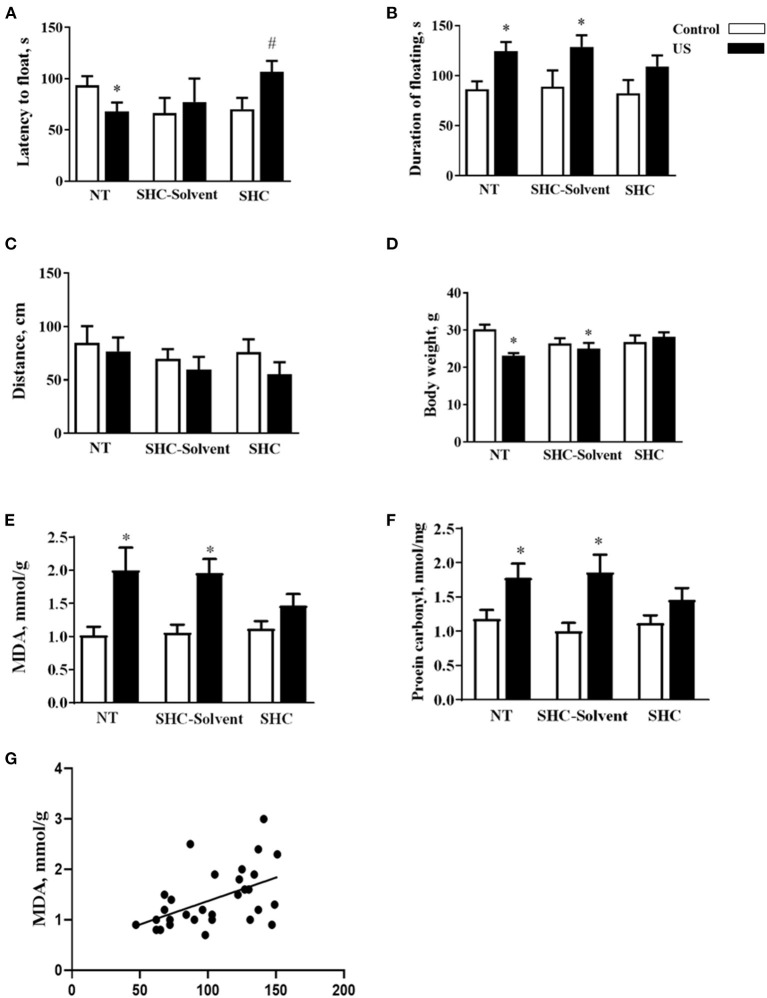
Effects of herbal antioxidant treatment on behavioral changes and oxidative stress markers of BALB/c mice exposed to US. In comparison with controls, US nontreated and SHC solvent–treated mice showed **(A)** shortened latency to float, **(B)** a significant prolongation of the duration of floating that was now shown for SHC-treated stressed mice. **(C)** No significant group differences were found in distance traveled in the open field. **(D)** Nontreated and SHC solvent–treated US groups had significant body weight loss in comparison with controls that was not shown for the SHC-treated stress group. Concentrations of **(E)** MDA and **(F)** protein carbonyl were higher in stressed nontreated and SHC solvent–treated groups than in controls, no such differences were shown for US SHC-treated mice. MDA concentration in the prefrontal cortex was significantly correlated with **(G)** the duration of floating and **(H)** body weight. *p < 0.05 vs. nontreated nonstressed controls, ^#^*p* < 0.05 vs. the nontreated stressed (US) group, two-way ANOVA and *post-hoc* Tukey's test. NT, nontreated. Bars are mean ± SEM.

Two-way ANOVA revealed significant effects of stress and treatment and no effect of their interaction on the latency to float in BALB/c mice subjected to the US (*F* = 5.16, *p* = 0.043; *F* = 4.94, *p* = 0.039; and *F* = 2.61, *p* = 0.936, respectively; two-way ANOVA). There was a significant effect of stress but not treatment or their interaction on the total duration of floating (*F* = 13.0, *p* = 0.001; *F* = 0.666, *p* = 0.523; and *F* = 0.180, *p* = 0.837, respectively). In comparison with controls, US-nontreated mice showed shortened latency to float (*p* = 0.038, Tukey's test; Figure 2A). In comparison with the latter group, this measure was significantly longer in the stressed SHC-treated mice (*p* = 0.023). A significant prolongation of the duration of floating in comparison with control group was shown for stressed nontreated and SCH-solvent-treated animals (*p* = 0.026 and 0.032, respectively), but not for SHC-treated stressed mice (*p* = 0.998, Figure 2B).

No significant group differences were found in distance traveled in the open field, and there was no effect of treatment, stress, or their interaction (*F* = 2.28, *p* = 0.141; *F* = 1.33, *p* = 0.28; and *F* = 0.376, *p* = 0.69, respectively; two-way ANOVA, Figure 2C). Significant effects of stress and stress × treatment interaction, but not treatment effect, were found for body weight (*F* = 4.3, *p* = 0.049; *F* = 4.76, *p* = 0.018; and *F* = 0.843, *p* = 0.442, respectively). Nontreated and SHC solvent–treated US groups had significant body weight loss in comparison with controls that was not shown for the SHC-treated stress group (*p* = 0.025, 0.048, and 0.791, respectively; Figure 2D). Together, the administration of SHC but not SHC solvent prevented US-induced despair behavior and loss of body mass that are classic signs of helplessness and stress. At the same time, US did not affect general locomotion, a potential source of artifacts in the evaluation of rodent behavior that rules out possible confounds in measuring helplessness in the present work.

There was a significant stress effect on MDA concentration although treatment and their interaction did not significantly affect this parameter (*F* = 19.2, *p* = 0.001; *F* = 1.68, *p* = 0.208; and *F* = 2.71, *p* = 0.086, respectively; two-way ANOVA). MDA concentration was higher in stressed nontreated and SHC solvent–treated groups than in controls (*p* = 0.001 and *p* = 0.002, Tukey's test; respectively), no such difference was shown for US SHC-treated mice (*p* = 0.092; Figure 2E). Similarly, we found a significant stress effect on the concentration of protein carbonyl (*F* = 16.5, *p* = 0.01); however, treatment and their interaction did not alter this measure (*F* = 1.55, *p* = 0.263 and *F* = 2.04, *p* = 0.093, respectively). The concentration of protein carbonyl was higher in stressed nontreated and SHC solvent–treated groups than in control mice (*p* = 0.01 and 0.01, respectively). No such difference was shown for US SHC-treated mice (*p* = 0.165; Figure 2F). MDA concentration in the prefrontal cortex was significantly correlated with the duration of floating (*p* = 0.002, *r* = 0.53; Pearson correlation, Figure 2G). There was a significant correlation between MDA levels and body weight (*p* = 0.008, *r* = −0.430). No significant correlation was shown between MDA concentration and the latency to float (*p* = 0.673, *r* = −0.079) and distance covered in the open field (*p* = 0.453, *r* = −0.014). As for protein carbonyl samples that were pulled together, no correlation analysis between this parameter of oxidative stress and behavior could be done. Collectively, the US-induced effects on behavior and oxidative stress markers were ameliorated by herbal antioxidant treatment.

### Herbal Antioxidant Treatment Normalizes Altered Behaviors and Pro-inflammatory and Distress Markers in C57BL/6 Mice Exposed to US

**Figure 3:**
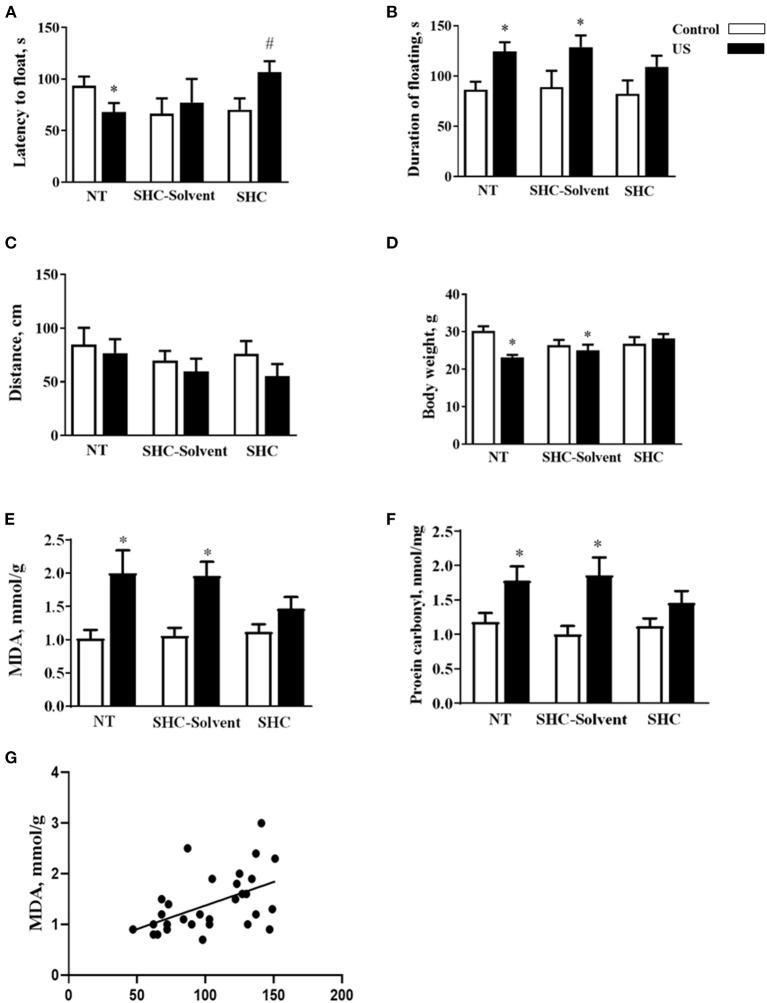
Ultrasound exposure of C57BL/6 mice induces abnormal behaviors and neuroinflammation: changes that are prevented by herbal antioxidant treatment. **(A)** Mice exposed to US that received no treatment but not stressed SHC-treated mice showed a significant prolongation of this behavior in comparison with controls. **(B)** In the open field, the number of rearings was significantly lower in nontreated US mice but not in US SHC-treated than in control. In the elevated O-maze, **(C)** the time spent in the open arms and **(D)** the number of exits therein were decreased in US nontreated animals in comparison with controls that was not found in US SHC-treated mice. **(E)** Several markers of inflammation and distress were studied for their gene expression in the prefrontal cortex. US-induced gene overexpression of IL-6 and IL-1β was ameliorated by SHC administration. **(F)** We found that US-exposed mice display the upregulation of several markers of inflammation and distress on a protein level. The overexpression of IL-6 and IL-1β, but not GSK-3α was normalized in the US SHC mice *p < 0.05 vs. nontreated nonstressed controls, #*p* < 0.05 vs. nontreated stressed group, one-way ANOVA and *post-hoc* Tukey's test. **(G)** The concentration of MDA significantly correlated with the duration of floating behaviour. Con, control nonstressed nontreated group. Bars are mean ± SEM.

C57BL/6 mice displayed significant group differences in the duration of floating (*F* = 3.691, *p* = 0.043, one-way ANOVA). Nontreated US mice but not stressed SHC-treated mice showed a significant prolongation of this behavior in comparison with controls (*p* = 0.023 and 0.695, respectively, Tukey's test; Figure 3A). In the open field, the number of rearings was significantly different between the groups (*F* = 3.569, *p* = 0.046); this score was significantly lower in nontreated US mice but not in US SHC-treated than in controls (*p* = 0.049 and 0.575, respectively; Figure 3B). No group differences in the distance traveled were found (*F* = 1.88, *p* = 0.201; *data not shown*).

In the elevated O-maze, there were significant group differences in the time spent in the open arms and the number of exits therein (*F* = 4.174, *p* = 0.036 and *F* = 5.961, *p* = 0.012, respectively; one-way ANOVA). In comparison with controls, US nontreated animals had a decrease in these measures (*p* = 0.018 and 0.032, respectively; Tukey's test) that was not found in US SHC-treated mice (*p* = 0.094 and 0.105, respectively, Figures 3C,D). Additionally, increased latency of risk assessment events and decreased number of these events were found in comparison with controls in the nontreated US group (*p* = 0.044 and 0.010, respectively, *data not shown*). These changes were not revealed in the SHC-treated group (*p* = 0.238 and 0.147, *data not shown*). These findings suggest increases of behavioral despair, anxiety-like behavior, and a decrease in the novelty exploration in C57BL/6 mice exposed to the US that were not observed in SHC-treated animals and are similar to above-described effects in the BALB/c strain.

There were significant group differences in the mRNA concentrations of IL-6 and IL-1β (*F* = 4.92, *p* = 0.018 and *F* = 4.12, *p* = 0.032, respectively; one-way ANOVA), but not of GSK-3β, GSK-3α, Akt, and IL-15 (*p* > 0.05). In comparison with controls, there was a significant increase of mRNA concentrations of IL-6 and IL-1β in the stressed nontreated mice (*p* = 0.015 and 0.032, respectively) but not in the stressed SHC-treated group (*p* = 0.638 and 0.33, respectively; Figure 3E). Results of the Western blot assay support most of the gene expression findings, showing significant group differences in the expression of IL-6 and IL-1β (*F* = 4.137, *p* = 0.037 and *F* = 4.22, *p* = 0.047, respectively) and a significant increase of both parameters in stressed nontreated mice but not in the stressed SHC-treated animals in comparison with controls (*p* = 0.029, 0.041 and 0.276, 0.85, respectively, Figure 3F). Significant group differences were found in protein levels of GSK-3α (*F* = 6.303, *p* = 0.030) that were increased in both nontreated and SHC-treated stressed mice in comparison with controls (*p* = 0.027 and 0.008, respectively).

Furthermore, the concentration of IL-1β mRNA significantly correlated with the duration of floating and the number of risk assessment events in the elevated O-maze (*p* = 0.039, *r* = 0.504 and *p* = 0.035, *r* = 0.534, respectively). Gene expression of two molecules with antistress functions, IL-15 and Akt, was significantly correlated with the duration of floating behavior (*p* = 0.025, *r* = 0.654 and *p* = 0.042, *r* = 0.409, respectively). Besides this, GSK-3α gene expression positively correlated with floating behavior (*p* = 0.015, *r* = 0.563) and negatively correlated with the time spent in the open arms of the O-maze (*p* = 0.01, *r* = −0.588). The concentration of MDA significantly correlated with the duration of floating behaviour (*p* = 0.021, *r* = 0.624; Figure 3G). Together, correlation analysis suggests that pro-inflammatory and stress-related changes in their prefrontal cortex are interrelated with altered behaviors in C57BL/6 mice subjected to the employed model. This highlights the significance of the improvement of both molecular and behavioral effects of the US by applied herbal antioxidant treatment. As for Western blot results, because samples were pulled together, the correlation analysis between this parameter and other readouts could not be done.

### Enhanced Learned Helplessness and Increases in Markers of Oxidative Stress in the modFST Are Prevented by the Administration of Herbal Antioxidant

**Figure 4:**
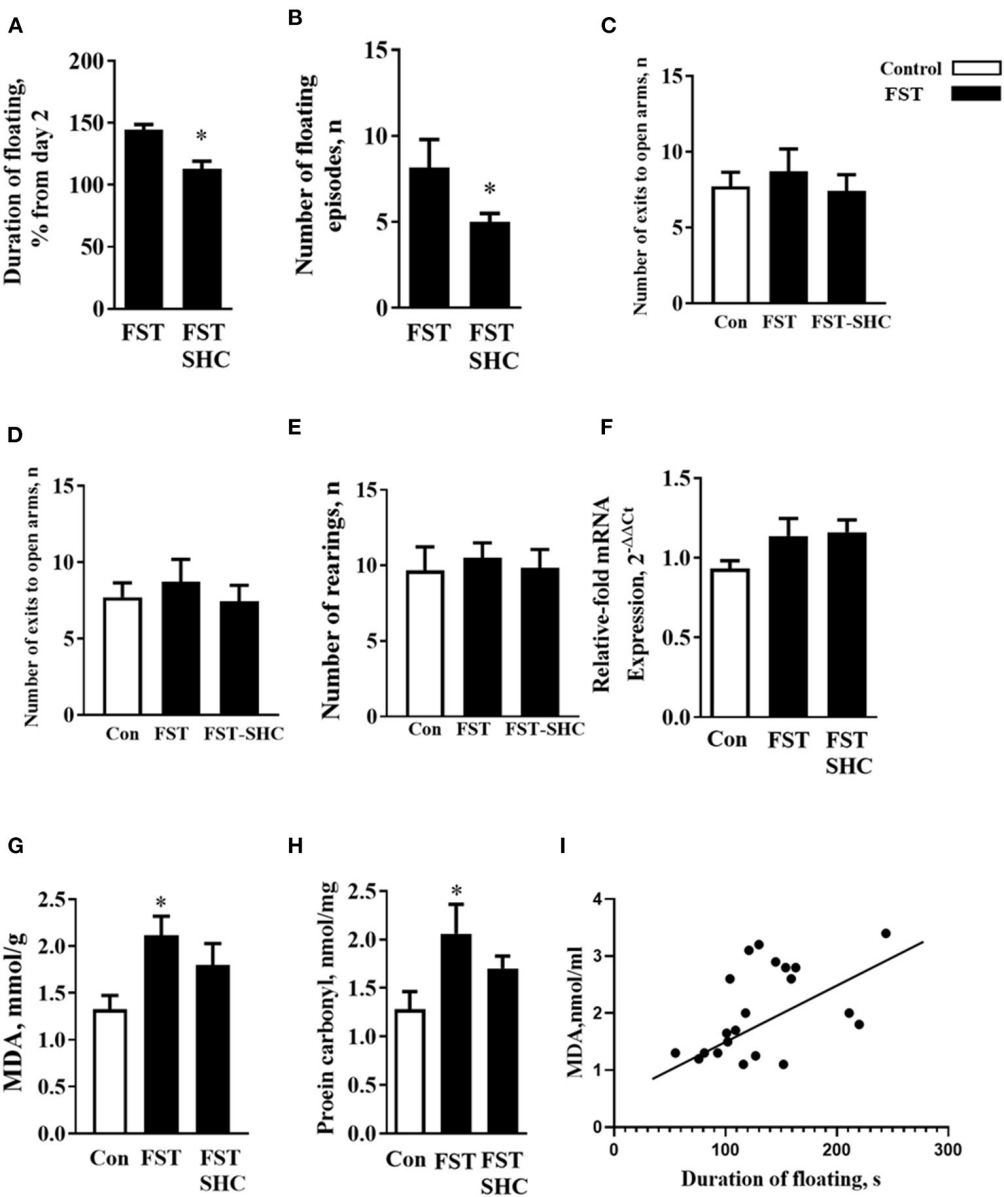
Oxidative stress and helplessness in the modFST are counteracted by herbal antioxidant treatment in C57BL/6 mice. In comparison to nontreated animals, **(A)** the duration of floating and **(B)** the number of floating episodes on day 5 normalized to day 2 were significantly lower in the SHC-treated mice; thus, the latter group showed a reduced potentiation of floating behavior by the end of repeated testing in the modFST. Mice exposed to the modFST revealed no significant differences in the **(C)** number of exits in the O-maze, **(D)** time spent therein, and **(E)** number of rears in the open field. **(F)** A three-group comparison that additionally included control naïve swimming mice revealed no significant group differences for mRNA GSK-3β concentrations but for normalized brain MDA concentration. **(G)** This measure was significantly higher in nontreated modFST-exposed animals than in those naïve for modFST. No such increase was shown by the SHC-treated modFST group, which had significantly lower MDA concentrations than the nontreated modFST-exposed group. **(H)** The level of protein carbonyl was increased in the nontreated modFST mice but not in the SHC-treated animals. **(I)** Significant correlation was found between the duration of floating on day 5 and MDA concentrations (Pearson correlation). *p < 0.05 vs. nontreated controls, *t*-test, or one-way ANOVA and Tukey's test (*see the ms text*). Bars are mean ± SEM.

The duration of floating and the number of floating episodes on day 5 normalized to day 2 were significantly lower in the SHC-treated group than in nontreated mice (*p* = 0.019 and 0.405, *t-*test; Figures 4A,B). Behavioral analysis of mice exposed to the modFST revealed a lack of group differences in the number of exits in the O-maze (*F* = 0.32, *p* = 0.725, one-way ANOVA), time spent therein (*F* = 0.39, *p* = 0.67), and number of rearings in the open field (*F* = 0.11, *p* = 0.88), suggesting that repeated swimming did not induce general effects on anxiety and novelty exploration (Figures 4C–E). A three-group comparison that additionally included control naïve for swimming mice revealed no significant group differences for mRNA GSK-3β concentrations (*F* = 3.109, *p* = 0.059, *F* = 7.91, *p* = 0.006; Figure 4F).

One-way ANOVA showed significant group differences in the MDA concentration (*F* = 7.91, *p* = 0.006) that was significantly higher in nontreated modFST-exposed animals than in naïve controls (*p* = 0.021, Tukey's test; Figure 4G). No such increase was observed within the SHC-treated modFST group (*p* = 0.87), which had significantly lower MDA concentrations than the nontreated modFST-exposed group (*p* = 0.006). For the level of protein carbonyl, similar group differences and increases in the nontreated modFST mice were found (*F* = 6.22, *p* = 0.04, 0.031, Figure 4H) that were not revealed in the SHC-treated animals exposed to this paradigm (*p* = 0.77). Significant correlation was found between the duration of floating on day 5 and MDA concentrations (*p* = 0.007, *r* = 0.65; Pearson correlation). No significant correlations were revealed between MDA levels and three behavioral parameters that were additionally investigated in modFST mice; the number of exits to the open arms of the elevated O-maze, time spent therein, and the number of rearings in the open field (*p* = 0.56, *r* = 0.133; *p* = 0.569, *r* = 0.140; and *p* = 0.723, *r* = 0.89, respectively). As for protein carbonyl samples that were pulled together, no correlation analysis between this parameter of oxidative stress and behavior could be done. Hence, these data suggest that helplessness in the modFST model correlates with the concentration of MDA in the prefrontal cortex of mice, and both of them can be prevented by a chronic pretreatment of herbal antioxidant used in this study (Figure 4I).

## Discussion

Our results are similar for the US and the modFST models' increases of the MDA levels in the prefrontal cortex and floating scores that significantly intercorrelate in each paradigm and were prevented by an antioxidant treatment with SHC. Protein carbonyl levels were increased in the prefrontal cortex of the US- and modFST-exposed mice, further suggesting comparable oxidative stress changes in these models. Significant correlations were also found between MDA concentrations and a loss of body weight in the US-exposed mice, additionally supporting a validity of brain MDA as an important marker of systemic stress response. Moreover, gene expression of key inflammatory and stress-related hallmarks, the IL-1 beta, IL-6, IL-15, Akt, and GSK-3 that are functionally related to oxidative stress significantly correlated with measures of floating behavior, anxiety-like changes, and disrupted novelty exploration in the US-exposed mice. Changes in gene expression of investigated molecular markers were in parallel with their protein expression changes. No alterations were found in anxiety and open field behaviors in the modFST model, nor significant correlations of these parameters with oxidative stress measures were revealed. Chronic administration of antioxidant treatment normalized the majority of stress-related behavioral and molecular changes in both mouse paradigms. Together, our data suggest that increased MDA accumulation and oxidative stress in the prefrontal cortex may mirror the overlapping features of MD- and PTSD-like syndromes that can be targetable by the same antioxidant therapies, such as SHC.

Extensive literature suggests the therapeutic potential of the components of SHC during stress-related disorders investigated here. For example, clove, *Syzygium aromaticum*, is used in traditional medicine to treat upset stomach, nausea, and inflammation of the mouth and throat (67). The bell pepper, *Capsicum annuum*, is shown to be enriched with antioxidants, such as vitamin C and E, provitamin A, ascorbic acid, and carotenoids (4). Basil, *Ocimum basilicum*, contains important antioxidants, such as p-coumaric acid, ferulic acid, isoquercitrin, rutin, and quercitrin (68). Likewise, pomegranate, *Punica granatum*, is also rich in antioxidants, e.g., ellagitannins, ellagic acids, anthocyanins, flavonols, flavan-3-ols, and flavones (3). Turmeric, *Curcuma longa*, is enriched with curcuminoids, including curcumin, a well-demonstrated antioxidant with anti-inflammatory and immunotropic properties (69). Finally, nettle, *Urtica dioica*, contains such important nutrients as terpenoids, β-carotene, neoxanthin, violaxanthin, lutein, lycopene, palmitic acids, cis-9,12-linoleic and α-linolenic acids, polyphenols, essential amino acids, and others (70).

Gas chromatography mass spectrometry analysis of the sample reveals that, apart from three monosaccharides used for gustatory properties of SHC, six elements constitute the SHC at concentrations exceeding 1% of the total dry weight of the sample. Among them is D-ribofuranose, which accounted for 2.5% of the total dry weight of the sample and which exists as two enantiomers: alpha-D-ribofuranose (aDR) and beta-D-ribofuranose (bDR). They are previously reported to modulate oxidative stress, immunity, and gut and microbiome functions and, thus, are likely to be active antistress elements of the investigated herbal antioxidant treatment (see Table 3). The main effects of these six elements are reviewed in a Supplementary Material.

**Table 3:** Functions of SHC chemical ingredients (for References, see Supplementary Material).

**Ingredient**	**Effects/roles**
Methyl α-D-Glucopyranoside	This is a non-metabolizable glucose analog (López-Yoldi et al., 2016; Veyhl-Wichmann et al., 2016), commercially exploited in food industry, biologically inactive in low amounts, broadly used for gustatory properties or/and crystallizing and surfactant agents in food industry
Methyl β-D-Galactopyranoside	It is used in food industry, has no known effect for human organism might indirectly affect gut microbiome via its effects on *E. coli* and Lactobacillus (Sahin-Tóth et al., 2002; Mukai et al., 1998).
D-Fructofuranose	It is used in food industry as the sweetener (Malik et al., 2015)
D-Ribofuranose	Its derivatives exhibit immunostimulatory, antinociceptive, anti-inflammatory, and potential anti-cancer effects (Petrelli et al., 2017; Ota et al., 2018; Rahman et al., 2020).
β-D-Lactose	IT induces fiber-like effect (Schaafsma, 2008), enhances intestinal mineral absorption particularly on calcium and magnesium (Abrams et al., 2002)
D-Glucose	It is present nearly in all plants, in low concentrations glucose does not induce any specific regulatory effects (Mergenthaler et al., 2013)
Malic acid	It is involved in citric acid cycle and stimulates metabolism with simultaneous decrease in tissue respiration, can ameliorate cell metabolism during of hypoxia (Dunaev et al., 1988; Tang et al., 2013)
Glyceric acid	As a precursor of serine, it is essential for neuronal metabolism, including protein and nucleotide synthesis, neurotransmitter synthesis, and lipids as well as glycolysis regulation (Tabatabaie et al., 2010)
Citric acid	It is implicated in energy generation in cells and exerts anti-hypoxia effects in ischemic neurons and astrocytes, suggested to play neuroprotective role (Ying et al., 2002; Abdel-Salam et al., 2014).

Our results were obtained in previously validated models of MD and PTSD. The US model of MD is designed to mimic emotional/mental stress in humans, a prevalent form of stress leading to neuropsychiatric pathology in a clinic (71). This type of stress, also called “psychological” or “informational” stress, is defined as a response to adverse experiences of a nonmaterial nature. In mice, the US paradigm was initially established on BALB/c mice and is reported to increase aggressive behavior, anhedonia, and helplessness and enhance hippocampal expression of stress-related factors, including GSK3β; oxidative stress markers, such as a drop in glutathione level; decreased functionality of plasticity markers, e.g., AMPA receptor subunits (8, 47, 48) and cause other negative effects. Classic antidepressants and compounds with antioxidative properties, including thiamine compounds, are shown to prevent most of these changes (47, 52).

Present data corroborate earlier reported results. A dysregulation of oxidative stress in the prefrontal cortex of US-exposed mice is in line with previously reported signs of oxidative stress in the hippocampus of US-exposed mice as shown by decreased levels of total glutathione and elevated concentrations of MDA and protein carbonyl in this brain structure (48, 52). These changes and an associated increase of pro-inflammatory cytokines were normalized by antioxidant thiamine compounds (52, 72). The finding of augmented floating scores in the nontreated US group is consistent with previous results reported in the US model (47) and are generally supported by reports of a relationship between increases in helplessness and chronic stress (73, 74). The demonstration of a significant correlation between MDA and floating scores suggests a causal relationship between these phenomena.

Behavioral parameters of an anxiety-like state in the O-maze were increased in nontreated US-exposed animals and correlated with overexpression of IL-6, IL-1 beta, and downregulation of anti-inflammatory cytokine IL-15. These findings and a decrease in exploratory activity of the nontreated US mice in the open field support previous findings of elevated anxiety and pro-inflammatory changes in this paradigm, shown in the hippocampal formation (8, 48, 52) as well other literature establishing neuroinflammation as a mechanism of pathological anxiety (5, 73, 75, 76). Anxiogenic-like changes of US mice also correlate with the overexpression of GSK-3β, a molecular hub of distress, and downregulation of its functional antagonist Akt. Both of these factors are implicated in the regulation of oxidative stress (77) and the production of pro-inflammatory cytokines (78, 79).

Chronic treatment of mice with an applied antioxidant composition has precluded US-induced increases of helplessness, anxiety-like behavior, and neophobia as well as most of the reported molecular changes pointing to the role of oxidative stress inflammation processes in these behavioral aberrations. Multiple comparisons reveal a lack of significant stress-induced increases in MDA and protein carbonyl content as well as overexpression of pro-inflammatory cytokines and distress marker GSK-3β in the US-exposed mice that were treated with SHC. Measured readouts in the US-exposed animals that received SHC solvent did not significantly differ from the changes in the stressed nontreated group. The treatment of nonstressed mice with the herbal composition did not significantly alter the investigated behavioral and molecular parameters.

Previous studies demonstrate the ameliorative effects of compounds with antioxidant and anti-inflammatory properties on behavioral parameters during stress (48, 51, 55). Other studies reveal the anxiolytic-like effects of compounds with antioxidant properties under stress conditions (3, 5, 80). Moreover, a relationship between the antianxiety action and normalization of MDA content in the brain is demonstrated in mice (69). Although beneficial functional effects of the herbal composition are to be ascribed to its antioxidant and anti-inflammatory effects, the administration of its solvent resulted in partial amelioration of several measures, such as body weight and IL-6 expression. These effects were reminiscent of the effects of the herbal composition itself and are likely due to well-known antistress and anti-inflammatory effects of alcohol (81–83), its main ingredient acting as a solvent. As such, SHC solvent might produce additive beneficial effects in the action of the herbal cocktail in question. At the same time, the effects of the solvent observed here are an important limitation that has to be addressed in the future studies.

The present study reveals an overexpression of GSK-3α on a protein level in stressed nontreated mice and its even greater increase in stressed mice that received SHC. These data further demonstrate the previously reported implication of GSK-3α expression in stress-related processes (24, 49) analogous with the well-documented role of GSK-3β as a molecular hub of distress. Both GSK-3 isoforms, GSK-3α and GSK-3β, are independently implicated in the regulation of similar molecular and cellular functions, such as cell development, apoptosis, and the mechanisms of distress, although there is also cross-talk between them (84, 85). Biochemical evidence shows that GSK-3α is generally involved in fast and relatively short-lasting cellular events, whereas GSK-3β activities tend to be associated with delayed and longer-lasting changes (86). Although the functions of GSK-3α and GSK-3β in a cell may overlap, GSK-3α determines a number of processes that are specific for this kinase (87), such as formation of hippocampal neurons (88) and a role in plasticity (89). For example, GSK3α, but not GSK-3β, has been implicated in fast spine shape remodeling and plasticity in the hippocampus (90). Our studies show a decrease of GSK-3α expression in the prefrontal cortex of mice exposed to two-day forced swimming and an increase of this measure after three repetitive swim sessions in the modFST model, suggesting complex dynamics in its regulation during stress (49). Based on these and present data and the above-discussed roles of this molecule in brain plasticity and remodeling, one can speculate that the increase in GSK-3α expression in the present study mirrors its potential adaptive effects under stress conditions, which might be potentiated by SHC treatment. Other findings with stress and antioxidant therapies are in line with current observations: chronic administration of thiamine has resulted in increased hippocampal GSK-3α expression along with amelioration of despair behavior in mice subjected to repeated forced swimming in the modFST model (49). Of note, pharmacological manipulation of GSK-3α activity had greater therapeutic effects than the GSK-3β-targeting treatments on apoptosis, a factor counteracting synaptic plasticity during stress (91), which further supports speculations about the role of plasticity and remodeling underlying the potentially positive effects of GSK-3α on brain functions.

Another recently proposed model used in the current study to address the potential effects of SHC is the model of enhanced learning of adversities, modFST. In this paradigm, increased floating behavior and GSK-3β overexpression were exhibited during the delayed swim session and were found to be context-dependent (24). The increase of floating behavior during the delayed test on day 5 correlates with brain overexpression of GSK-3β that was validated as a biomarker of enhanced learning of adverse context. Both changes were associated with the exposure of animals to the context alone and were previously shown to be reversible by pretreatment with antidepressant and antioxidant thiamine compounds. In addition, increased floating behavior was accompanied by the overexpression of the pro-inflammatory mediators IL-1β, TNF, and COX-1 and increases in brain glutathione and protein carbonyl levels in the hippocampus and prefrontal cortex (49–51).

In line with previous studies showing that the majority of neurobiological abnormalities in the modFST are prevented by chronic pretreatment with administration of thiamine compounds (49, 50), the administration of SHC ameliorated protein oxidative stress markers in the prefrontal cortex. Importantly, the MDA levels in the prefrontal cortex were significantly correlated with the duration of floating behavior. Present findings in the modFST of an upregulation of MDA and protein carbonyl in the prefrontal cortex are in good agreement with previously reported results regarding antioxidant therapies. These data are in keeping with the literature suggesting compromised markers of oxidative stress during depression and comorbid disorders, such as PTSD, and their normalization by antidepressant treatment (11, 92). In the present study, we found no significant increases in the expression of GSK-3β; as a consequence, no significant changes in this measure were found in SHC-treated mice. This can be due to the confounding effects of intense handling during dosing of animals with antioxidant treatment that is a key element of contextual learning in the modFST model. In summary, our data, demonstrating ameliorating effects of SHC in modFST-exposed mice, together with the above-discussed previous findings further argue for the importance of oxidative stress in the PTSD-like induced syndrome and the therapeutic potential of herbal antioxidant therapy during this condition.

## Conclusions

Thus, the continual administration of SHC to mice during their exposure to the emotional US model or to a paradigm of enhanced learning of adversities/PTSD results in normalizing effects on the measures of behavioral despair and anxiety and the concentration of oxidative stress markers. The comparison of neurochemical changes in the prefrontal cortex and behavioral changes in two novel mouse models of MD and PTSD reveals an intricate overlap between oxidative stress and helplessness. Thus, analogous with shared antidepressant drug therapy used in patients suffering from these disorders and their comorbidities, there is a potential that the use of antioxidant remedies, such as SHC, can be equally effective in both of these conditions. Several pharmacokinetics and pharmacodynamics analyses have to be carried out before comparing the efficacy of SHC with standard antidepressants and bringing it to patients. It has to be noted that the average efficacy of antidepressants that are mainly used in therapy of PTSD comorbid with MD is about 60%, whereas for placebo, it approaches 40% (11). Given this limited response rate, herbal treatments may be considered as effective and safe supplements to current medications of PTSD comorbid with MD and other stress-related disorders. As medicinal herbs exert fewer side effects than conventional drugs and are affordable for low-income societies (93), the use of such therapies appears to be particularly beneficial for the improvement of mental health under conditions of the ongoing economic and medicinal crisis related to the COVID-19 pandemic.

## Data Availability Statement

The raw data supporting the conclusions of this article will be made available by the authors, without undue reservation.

## Ethics Statement

The animal study was reviewed by Zuyderland Zuid, METC the Netherlands and approved by Ethical committee of MSMU (07-2019).

## Author Contributions

CS, TS, and JM conceived the study. MS, AK, AU, and AS designed the experiments. DP, AG, AN, and AP carried out the animal experiments. JM, DP, and AG performed the molecular analyses. JM, TS, AU, and AS performed data analysis. K-PL, AS, and TS supervised the project. K-PL, TS, and AS got the funding. CS, JM, DK, MS, and TS wrote the initial draft of the manuscript and all other authors listed here revised it. All authors contributed to the article and approved the submitted version.

## Conflict of Interest

The authors declare that the research was conducted in the absence of any commercial or financial relationships that could be construed as a potential conflict of interest.
