# Supplementary material for: Increased Oxidative Stress in the Prefrontal Cortex as a Shared Feature of Depressive- and PTSD-Like Syndromes: Effects of a Standardized Herbal Antioxidant
Source: Front Nutr. 2021 Apr 15;8:661455. doi: 10.3389/fnut.2021.661455 (PMC8086427; doi:10.3389/fnut.2021.661455)
Supplement: Supplementary file 1 [file Data_Sheet_1.docx]

**Supplementary file**

**Supplementary Table 1. Sequences of primers used for qRT-PCR**

| **Gene** | **Forward primer 5′–3′** | **Reverse primer 5′–3′** |
| --- | --- | --- |
| GAPDH | TGCACCACCAACTGCTTAG | GGATGCAGGGATGATGTTC |
| GSK3α | AATCTTGGCCAGTCTGAGCT | TCAGTCCTGGTGAACTGTCC |
| GSK3β | TCCATTCCTTTGGAATCTGC | CAATTCAGCCAACACACAGC |
| Akt | GGCTGCTCAAGAAGGACCCTAC | GGTGCTGCATGATCTCCTTGG |
| IL-1β | TGTAATGAAAGACGGCACACC | TCTTCTTTGGGTATTGCTTGG |
| IL-6 | ACTCACCTCTTCAGAACGAATTG | CCATCTTTGGAAGGTTCAGGTTG |
| IL-15 | CCATCCAGTGCTACTTGTGTTTAC | CCAGTTGGCTTCTGTTTTAGGAA |

**Supplementary** **Table 2. Primary antibodies used in the Western blot assay**

| **Antibody** | **Dilution** |
| --- | --- |
| Anti-β-tubulin (Abcam, Cambridge, MA, USA) Ab8227 | 1:700 |
| Anti-IL-6 (Abcam, Cambridge, MA, USA) Ab208113 | 1:1000 |
| Anti-GSK-3β (Abcam, Cambridge, MA, USA) Ab32391 | 1:1000 |
| Anti- GSK-3a (Abcam, Cambridge, MA, USA) Ab40870 | 1:1000 |
| Anti-IL-1β (Abcam, Cambridge, MA, USA) Ab9722 | 1:1000 |
| Anti-IL-15 (Abcam, Cambridge, MA, USA) Ab7213 | 1:500 |
| Anti-AktpSer473 (Abcam, Cambridge, MA, USA) Ab81283 | 1:5000 |

**Gas chromatography mass-spectrometry analysis of SHC**

Using extraction of derivatives, gas chromatography mass-spectrometry (GC-MS) analysis of SHC was carried out.

*Extraction with ethyl acetate*. 100 μl of SHC was placed into a glass vial with a screw cap, 1.0 ml of ethyl acetate (test material / solvent ratio = 1/10) was added, the mixture was thoroughly vortexed. The vial is placed on a hotplate and heated at 50° C for 24 hours. Thereafter, 200 μl of solution was centrifuged at 12000 rpm (4° C) for 10 min. Supernatant was used for further analyses.

*Extraction of derivatives*. 20 μL of a mixture of N,O-bis (trimethylsilyl) trifluoroacetamide (BSTFA) and trimethylchlorosilane (TMSC) (BSTFA to TMSC ratio was 99:1) was added to 20 μL of the supernatant. This mixture was placed on the hotplate at 75° C for 60 minutes. 70 μl of ethyl acetate was added, stirred and used for further analyses.

The GC-MS analysis was performed on a Chromatek GC-MS analyzer, consisted of a Chromatek-Kristall 5000 gas chromatograph and a DAZH-2M (3D) dispenser (Chromatek ltd., Yoshkar-Ola, Russia). Capillary column Phenomenex ZB-DRUG-1 (Phenomenex ltd., Aschaffenburg, Germany) with parameters: 30 m x 0.25 mm x 0.25 μm was used. Mass-spectrometry detector conditions were: stream division 5.0; ion source temperature 200°C; transition line temperature 290°С; scanning range = 50-550 atomic mass unit; sample volume 1 μl. For the identification of derivatives, an automatic database gas chromatography-mass spectrometry NIST14 MS Library (Adaptas SIS Ltd., Palmer, MA 01069. USA) was used. The main results of gas chromatography mass-spectrometry analysis are presented in a Table 4 of the main text.

# Summary of reported physiological effects of SHC main chemical components

Chemical analysis of the sample via GC–MS revealed prevalence of three monosaccharides: alpha-methylglucoside (39.4%), methyl beta-galactoside (9.5%), and fructofuranose (5.5%) – all of which are commercially exploited in food industry, and in low amounts are overly biologically inactive. These monosaccharides are broadly used for gustatory properties or / and crystallizing and surfactant agents in food industry that is approved by the FDA (Food and Drug Administration of the USA). Particularly, alpha-methylglucoside (aMG) is known to be a non-metabolizable glucose analog (López-Yoldi et al., 2016; Veyhl-Wichmann et al., 2016), acting as a reward for flavor preference in mice (Zukerman et al., 2013). As for methyl beta-galactoside (MbG), its physiological role in humans is not proven, while indirect effects MbG via gut microbiome can be suggested by the data showing its metabolic role in Escherichia coli and Lactobacillus (Mukai et al., 1998; Sahin-Tóth et al., 2002). In humans, D-fructofuranose (also known as fructose), is metabolized almost completely in the liver, it does not serve as a primary source of energy being is predominantly transformed into triglycerides and fatty acids (Mayes, 1993) and used in food industry as the sweetener (Malik and Hu, 2015).

Apart from three monosaccharides that were found to be the most abundant components of the SHC, six elements were found in the SHC at the concentrations exceeding 1% of the total dry weight of the sample. Among them is D-ribofuranose, which accounted for 2.5% of the total dry weight of the sample, which exists as two enantiomers: alpha-D-ribofuranose (aDR) and beta-D-ribofuranose (bDR). aDR has been widely utilized for synthesizing nucleotide and nucleoside analogs with a wide spectrum of biological effects. For example, some aDR derivatives demonstrated a powerful antinociceptive effect in mice (Petrelli et al., 2017). In line with these findings, Rahman et al. have discovered analgesic and anti-inflammatory activities in several aDR-based substances (Rahman et al., 2020). Nucleoside analogs have also been widely used in cancer chemotherapy as inhibitors of enzymes involved in intracellular nucleoside metabolism (Galmarini et al., 2002). bDR was suggested to exert immunostimulatory properties and was studied as an element of traditional Chinese medicine (Ota et al., 2019).

Another constituent of the sample was β-D-lactose (bDL), a disaccharide consisting of galactose and glucose, whose primary source is cow milk. The most recent comprehensive review by Schaafsma sums up all information available for lactose, highlighting its fiber-like activities and positive effects on absorption of minerals (Schaafsma, 2008), particularly on calcium and magnesium (Abrams et al., 2002).

The dry sample of SHC was constituting 2% of glucose, which can be found in most plant-based and animal products, either individually or, most frequently, incorporated into complex molecules, e.g., oligo- and polysaccharides. Low glucose content in the sample studied suggests its primary plant origin in the SHC and makes unlikely the possibility of any specific physiological effects of reported glucose amount on CNS or metabolism in a current study (Mergenthaler et al., 2013).

Malic acid (MA; 3.2% of the total dry weight of the sample) was postulated to show promising adaptogenic properties in 1988 by Dunaev et al. They discovered that this substance, found in various fruits, particularly citruses, promoted neuron excitation in sensory and motor brain areas in a dose-dependent manner (Dunaev et al., 1988). The observed effects were attributed to metabolism stimulation with simultaneous decrease in tissue respiration, since MA is involved in CAC (citric acid cycle), and introduction of MA into the cell thus replenishes the pool of substrate for further oxidation with less oxygen-dependent stages during the CAC. MA is also involved in malate-aspartate shuttle responsible for NADH transport across mitochondrial membrane utilized for energy production. Owing to its biological effects in energy-demanding cells, e.g., neurons and muscle cells, possible advantages of malic acid supplementation have been under investigation, generally yielding positive results (Bendahan et al., 2002; Qiang, 2015). Particularly curious results were obtained when studying the effects of MA in rat myocardial ischemia, where it exhibited cardiomyocyte-protective action, thus upholding conclusions made by other scholars (Tang et al., 2013).

Glyceric acid (GA) is a precursor of serine, an amino acid essential for neuronal metabolism, including protein and nucleotide synthesis, neurotransmitter synthesis and lipids (Tabatabaie et al., 2010). A deficiency of D-glycerate 2 kinase, an enzyme responsible for GA phosphorylation, has been associated with severe infantile epileptic encephalopathy as a result of D-glyceric acidemia (Zehavi et al., 2019). Additionally, some of GA’s phosphate derivatives (e.g., 2-phosphoglyceric acid, 3-phosphoglyceric acid, 2,3-bisphosphoglyceric acid, and 1,3-bisphosphoglyceric acid) are involved in glycolysis either directly or as regulatory molecules.

Much like MA, citric acid (CA) is involved in CAC and, therefore, energy generation in cells. CAC substrates have been shown to prevent death of ischemic neurons and astrocytes (Ying et al., 2002). Further studies by Abdel-Salam et al. have demonstrated CA’s neuroprotective effects in models of oxidative stress (Abdel-Salam et al., 2014) and malathion intoxication (Abdel-Salam et al., 2014). It is hypothesized that the underlying CA’s activities rely on the same mechanism that those of MA’s, following the logic of them both being involved in CAC. The summary of main Functions of SHC chemical ingredients in human physiology are presented in a Table 3 of the main text.

**Reference list**

Abdel-Salam, O.M.E., Youness, E.R., Mohammed, N.A., Morsy, S.M.Y., Omara, E.A., Sleem, A.A., 2014. Citric acid effects on brain and liver oxidative stress in lipopolysaccharide-treated mice. J Med Food 17, 588–598. https://doi.org/10.1089/jmf.2013.0065

Abrams, S.A., Griffin, I.J., Davila, P.M., 2002. Calcium and zinc absorption from lactose-containing and lactose-free infant formulas. Am J Clin Nutr 76, 442–446. https://doi.org/10.1093/ajcn/76.2.442

Bendahan, D., Mattei, J.P., Ghattas, B., Confort-Gouny, S., Le Guern, M.E., Cozzone, P.J., 2002. Citrulline/malate promotes aerobic energy production in human exercising muscle. Br J Sports Med 36, 282–289. https://doi.org/10.1136/bjsm.36.4.282

Costa-Nunes, J.P., Gorlova, A., Pavlov, D., Cespuglio, R., Gorovaya, A., Proshin, A., Umriukhin, A., Ponomarev, E.D., Kalueff, A.V., Strekalova, T., Schroeter, C.A., 2020. Ultrasound stress compromises the correlates of emotional-like states and brain AMPAR expression in mice: effects of antioxidant and anti-inflammatory herbal treatment. Stress 23, 481–495. https://doi.org/10.1080/10253890.2019.1709435

Dunaev, V.V., Tishkin, V.S., Milonova, N.P., Belaĭ, I.M., Makarenko, A.N., 1988. [Effect of malic acid salts on physical work capacity and its recovery after exhausting muscular activity]. Farmakol Toksikol 51, 21–25.

Galmarini, C.M., Mackey, J.R., Dumontet, C., 2002. Nucleoside analogues and nucleobases in cancer treatment. Lancet Oncol 3, 415–424. https://doi.org/10.1016/s1470-2045(02)00788-x

Gorlova, A., Pavlov, D., Anthony, D.C., Ponomarev, E.D., Sambon, M., Proshin, A., Shafarevich, I., Babaevskaya, D., Lesсh, K.-P., Bettendorff, L., Strekalova, T., 2019. Thiamine and benfotiamine counteract ultrasound-induced aggression, normalize AMPA receptor expression and plasticity markers, and reduce oxidative stress in mice. Neuropharmacology 156, 107543. https://doi.org/10.1016/j.neuropharm.2019.02.025

López-Yoldi, M., Castilla-Madrigal, R., Lostao, M.P., Barber, A., Prieto, J., Martínez, J.A., Bustos, M., Moreno-Aliaga, M.J., 2016. Cardiotrophin-1 decreases intestinal sugar uptake in mice and in Caco-2 cells. Acta Physiol (Oxf) 217, 217–226. https://doi.org/10.1111/apha.12674

Malatynska, E., Steinbusch, H.W.M., Redkozubova, O., Bolkunov, A., Kubatiev, A., Yeritsyan, N.B., Vignisse, J., Bachurin, S., Strekalova, T., 2012. Anhedonic-like traits and lack of affective deficits in 18-month-old C57BL/6 mice: Implications for modeling elderly depression. Exp Gerontol 47, 552–564. https://doi.org/10.1016/j.exger.2012.04.010

Malik, V.S., Hu, F.B., 2015. Fructose and Cardiometabolic Health: What the Evidence From Sugar-Sweetened Beverages Tells Us. J Am Coll Cardiol 66, 1615–1624. https://doi.org/10.1016/j.jacc.2015.08.025

Mayes, P.A., 1993. Intermediary metabolism of fructose. Am J Clin Nutr 58, 754S-765S. https://doi.org/10.1093/ajcn/58.5.754S

Mergenthaler, P., Lindauer, U., Dienel, G.A., Meisel, A., 2013. Sugar for the brain: the role of glucose in physiological and pathological brain function. Trends Neurosci 36, 587–597. https://doi.org/10.1016/j.tins.2013.07.001

Morozova, A., Zubkov, E., Strekalova, T., Kekelidze, Z., Storozeva, Z., Schroeter, C.A., Bazhenova, N., Lesch, K.-P., Cline, B.H., Chekhonin, V., 2016. Ultrasound of alternating frequencies and variable emotional impact evokes depressive syndrome in mice and rats. Prog Neuropsychopharmacol Biol Psychiatry 68, 52–63. https://doi.org/10.1016/j.pnpbp.2016.03.003

Mukai, T., Kaneko, S., Ohori, H., 1998. Haemagglutination and glycolipid-binding activities of Lactobacillus reuteri. Lett Appl Microbiol 27, 130–134. https://doi.org/10.1046/j.1472-765x.1998.00418.x

Ota, M., Ishiuchi, K., Xu, X., Minami, M., Nagachi, Y., Yagi-Utsumi, M., Tabuchi, Y., Cai, S.-Q., Makino, T., 2019. The immunostimulatory effects and chemical characteristics of heated honey. J Ethnopharmacol 228, 11–17. https://doi.org/10.1016/j.jep.2018.09.019

Pavlov, D., Bettendorff, L., Gorlova, A., Olkhovik, A., Kalueff, A.V., Ponomarev, E.D., Inozemtsev, A., Chekhonin, V., Lesсh, K.-P., Anthony, D.C., Strekalova, T., 2019. Neuroinflammation and aberrant hippocampal plasticity in a mouse model of emotional stress evoked by exposure to ultrasound of alternating frequencies. Prog Neuropsychopharmacol Biol Psychiatry 90, 104–116. https://doi.org/10.1016/j.pnpbp.2018.11.014

Pavlov, D., Gorlova, A., Bettendorff, L., Kalueff, A.A., Umriukhin, A., Proshin, A., Lysko, A., Landgraf, R., Anthony, D.C., Strekalova, T., 2020. Enhanced conditioning of adverse memories in the mouse modified swim test is associated with neuroinflammatory changes - Effects that are susceptible to antidepressants. Neurobiol Learn Mem 172, 107227. https://doi.org/10.1016/j.nlm.2020.107227

Pavlov, D., Markova, N., Bettendorff, L., Chekhonin, V., Pomytkin, I., Lioudyno, V., Svistunov, A., Ponomarev, E., Lesch, K.-P., Strekalova, T., 2017. Elucidating the functions of brain GSK3α: Possible synergy with GSK3β upregulation and reversal by antidepressant treatment in a mouse model of depressive-like behaviour. Behav Brain Res 335, 122–127. https://doi.org/10.1016/j.bbr.2017.08.018

Petrelli, R., Scortichini, M., Kachler, S., Boccella, S., Cerchia, C., Torquati, I., Del Bello, F., Salvemini, D., Novellino, E., Luongo, L., Maione, S., Jacobson, K.A., Lavecchia, A., Klotz, K.-N., Cappellacci, L., 2017. Exploring the Role of N6-Substituents in Potent Dual Acting 5’-C-Ethyltetrazolyladenosine Derivatives: Synthesis, Binding, Functional Assays, and Antinociceptive Effects in Mice ∇. J Med Chem 60, 4327–4341. https://doi.org/10.1021/acs.jmedchem.7b00291

Qiang, F., 2015. Effect of Malate-oligosaccharide Solution on Antioxidant Capacity of Endurance Athletes. Open Biomed Eng J 9, 326–329. https://doi.org/10.2174/1874120701509010326

Rahman, F.I., Hussain, F., Saqueeb, N., Abdur Rahman, S.M., 2020. Synthesis and evaluation of pharmacological activities of some 3-O-benzyl-4-C-(hydroxymethyl)-1,2-O-isopropylidene-α-D-ribofuranose derivatives as potential anti-inflammatory agents and analgesics. Res Pharm Sci 15, 209–217. https://doi.org/10.4103/1735-5362.288423

Sahin-Tóth, M., Gunawan, P., Lawrence, M.C., Toyokuni, T., Kaback, H.R., 2002. Binding of hydrophobic D-galactopyranosides to the lactose permease of Escherichia coli. Biochemistry 41, 13039–13045. https://doi.org/10.1021/bi0203076

Schaafsma, G., 2008. Lactose and lactose derivatives as bioactive ingredients in human nutrition. International Dairy Journal 18, 458–465. https://doi.org/10.1016/j.idairyj.2007.11.013

Strekalova, T., Anthony, D.C., Dolgov, O., Anokhin, K., Kubatiev, A., Steinbusch, H.M.W., Schroeter, C., 2013. The differential effects of chronic imipramine or citalopram administration on physiological and behavioral outcomes in naïve mice. Behav Brain Res 245, 101–106. https://doi.org/10.1016/j.bbr.2013.02.015

Strekalova, T., Evans, M., Chernopiatko, A., Couch, Y., Costa-Nunes, J., Cespuglio, R., Chesson, L., Vignisse, J., Steinbusch, H.W., Anthony, D.C., Pomytkin, I., Lesch, K.-P., 2015. Deuterium content of water increases depression susceptibility: the potential role of a serotonin-related mechanism. Behav Brain Res 277, 237–244. https://doi.org/10.1016/j.bbr.2014.07.039

Strekalova, T., Markova, N., Shevtsova, E., Zubareva, O., Bakhmet, A., Steinbusch, H.M., Bachurin, S., Lesch, K.-P., 2016. Individual Differences in Behavioral Despair Predict Brain GSK-3beta Expression in Mice: The Power of a Modified Swim Test. Neural Plast 2016, 5098591. https://doi.org/10.1155/2016/5098591

Tabatabaie, L., Klomp, L.W., Berger, R., de Koning, T.J., 2010. l-Serine synthesis in the central nervous system: A review on serine deficiency disorders. Molecular Genetics and Metabolism 99, 256–262. https://doi.org/10.1016/j.ymgme.2009.10.012

Tang, X., Liu, J., Dong, W., Li, P., Li, L., Lin, C., Zheng, Y., Hou, J., Li, D., 2013. The cardioprotective effects of citric Acid and L-malic Acid on myocardial ischemia/reperfusion injury. Evid Based Complement Alternat Med 2013, 820695. https://doi.org/10.1155/2013/820695

Veyhl-Wichmann, M., Friedrich, A., Vernaleken, A., Singh, S., Kipp, H., Gorboulev, V., Keller, T., Chintalapati, C., Pipkorn, R., Pastor-Anglada, M., Groll, J., Koepsell, H., 2016. Phosphorylation of RS1 (RSC1A1) Steers Inhibition of Different Exocytotic Pathways for Glucose Transporter SGLT1 and Nucleoside Transporter CNT1, and an RS1-Derived Peptide Inhibits Glucose Absorption. Mol Pharmacol 89, 118–132. https://doi.org/10.1124/mol.115.101162

Ying, W., Chen, Y., Alano, C.C., Swanson, R.A., 2002. Tricarboxylic acid cycle substrates prevent PARP-mediated death of neurons and astrocytes. J Cereb Blood Flow Metab 22, 774–779. https://doi.org/10.1097/00004647-200207000-00002

Zehavi, Y., Mandel, H., Eran, A., Ravid, S., Abu Rashid, M., Jansen, E.E.W., Wamelink, M.M.C., Saada, A., Shaag, A., Elpeleg, O., Spiegel, R., 2019. Severe infantile epileptic encephalopathy associated with D-glyceric aciduria: report of a novel case and review. Metab Brain Dis 34, 557–563. https://doi.org/10.1007/s11011-019-0384-x

Zukerman, S., Ackroff, K., Sclafani, A., 2013. Post-oral appetite stimulation by sugars and nonmetabolizable sugar analogs. Am J Physiol Regul Integr Comp Physiol 305, R840-853. https://doi.org/10.1152/ajpregu.00297.2013
